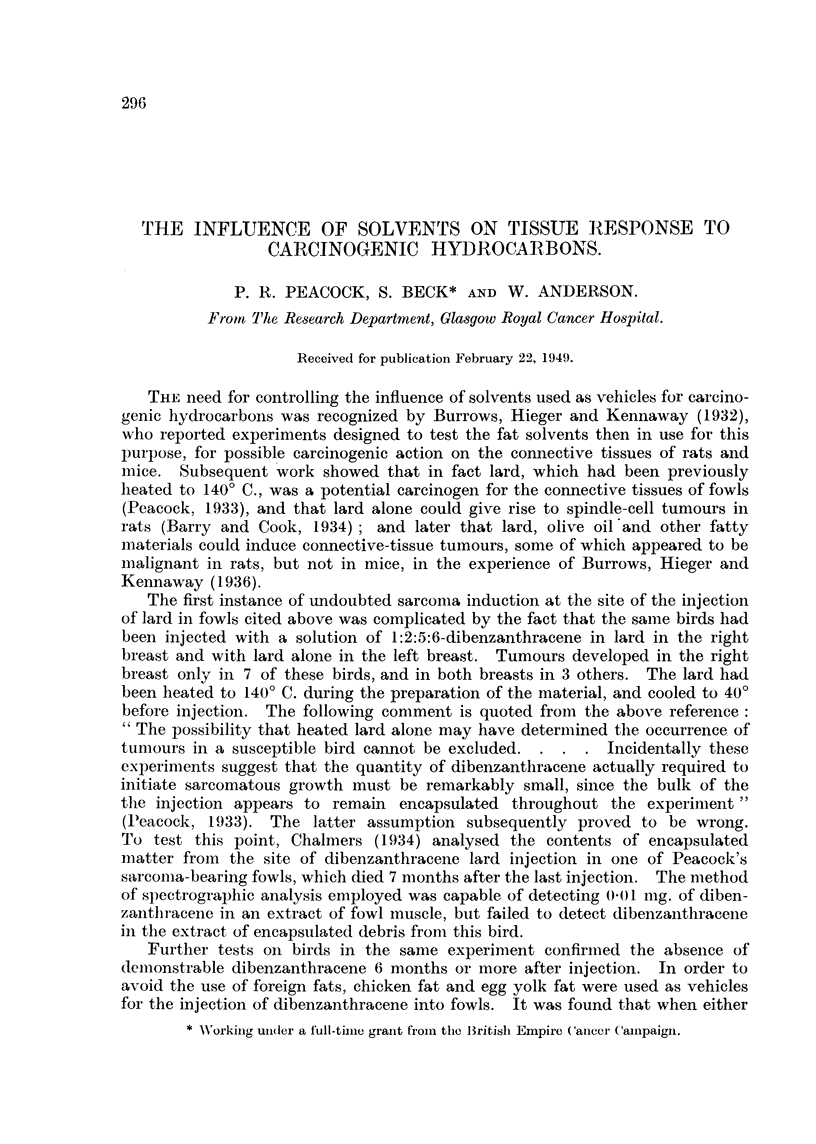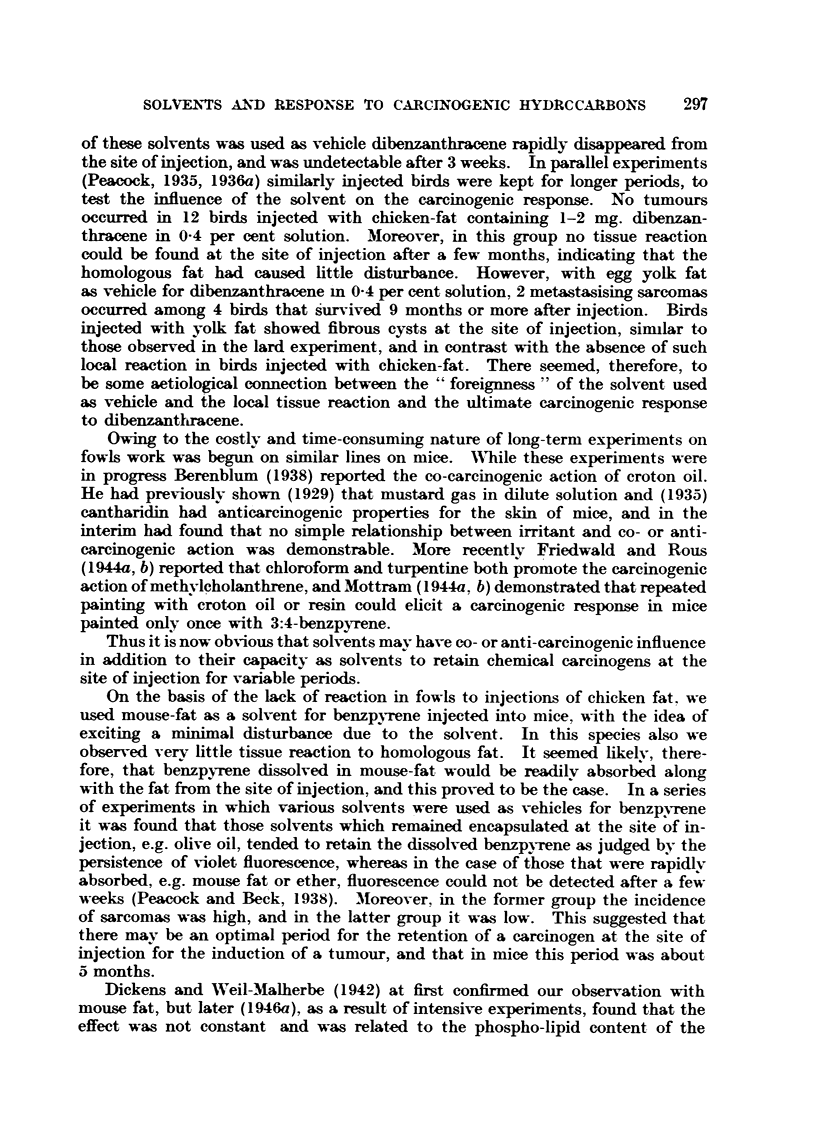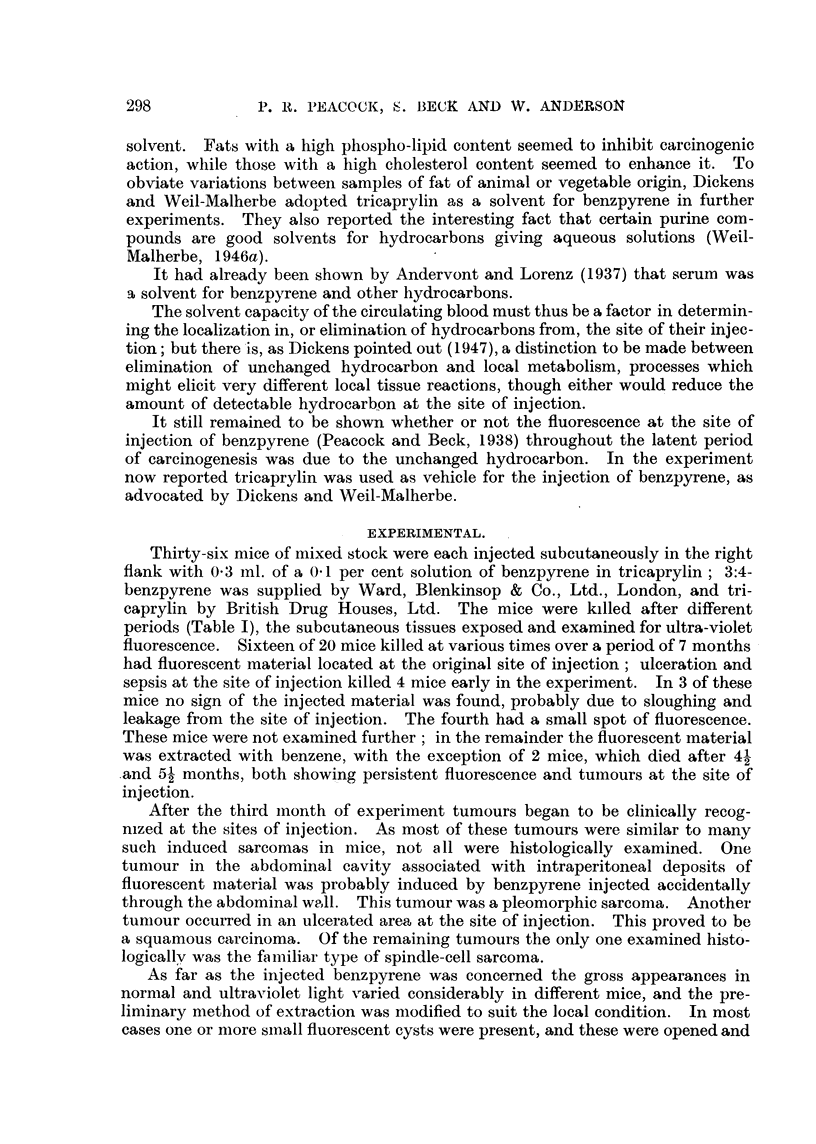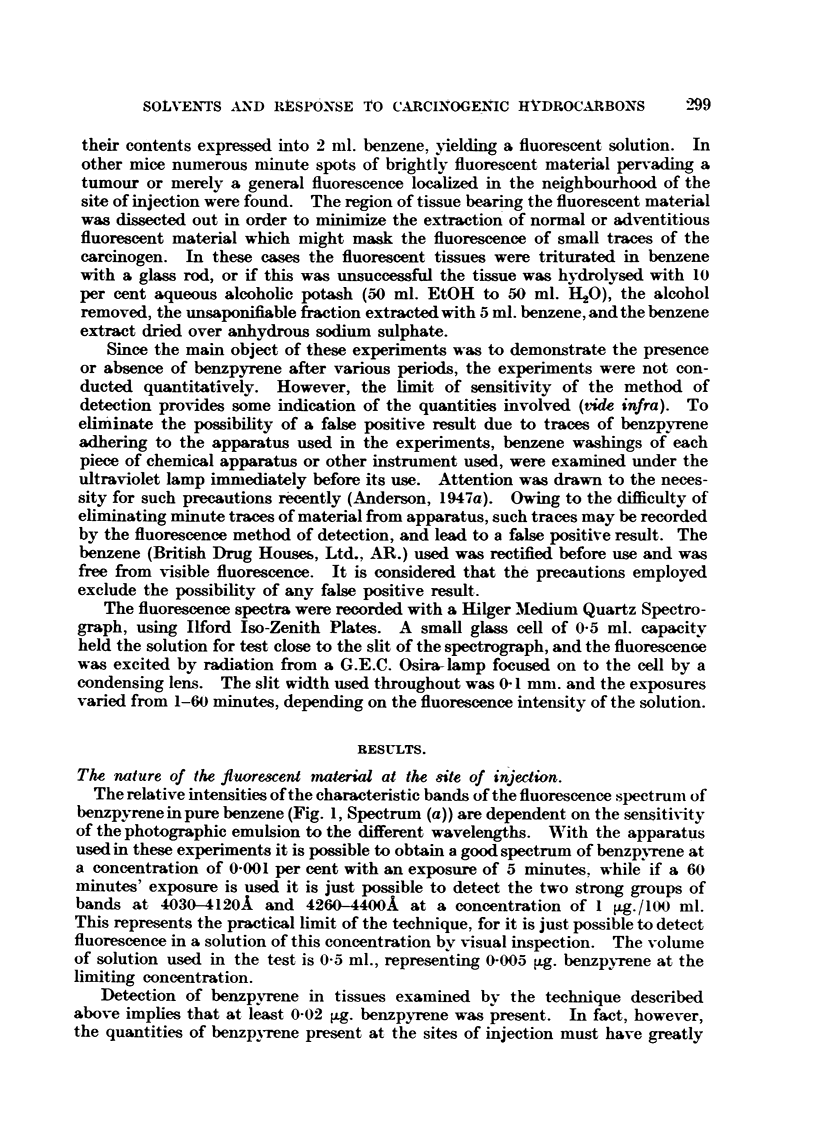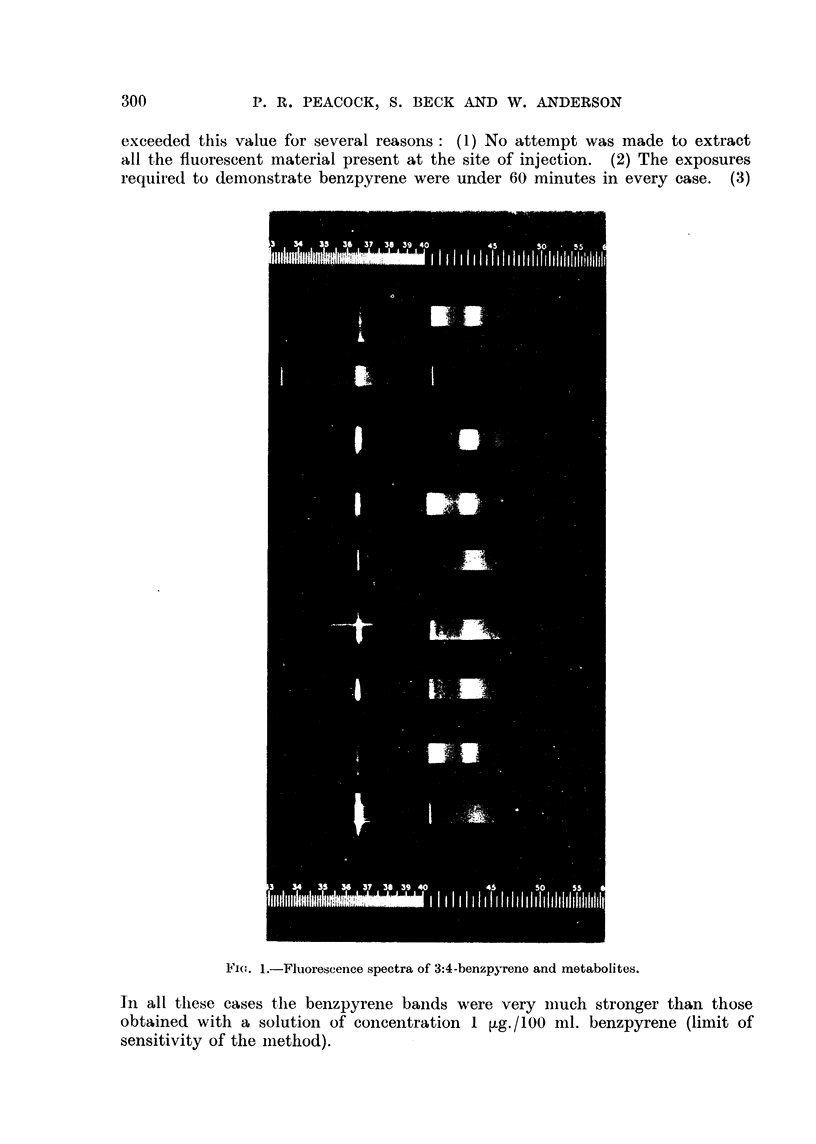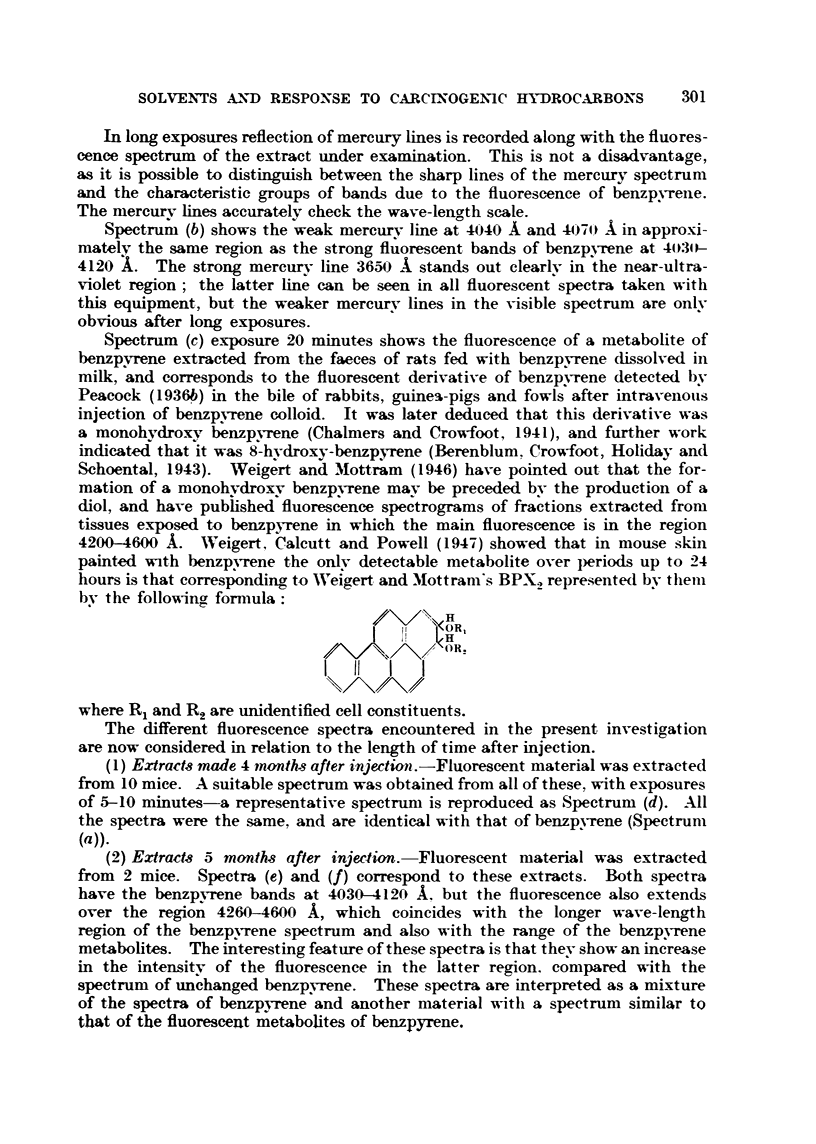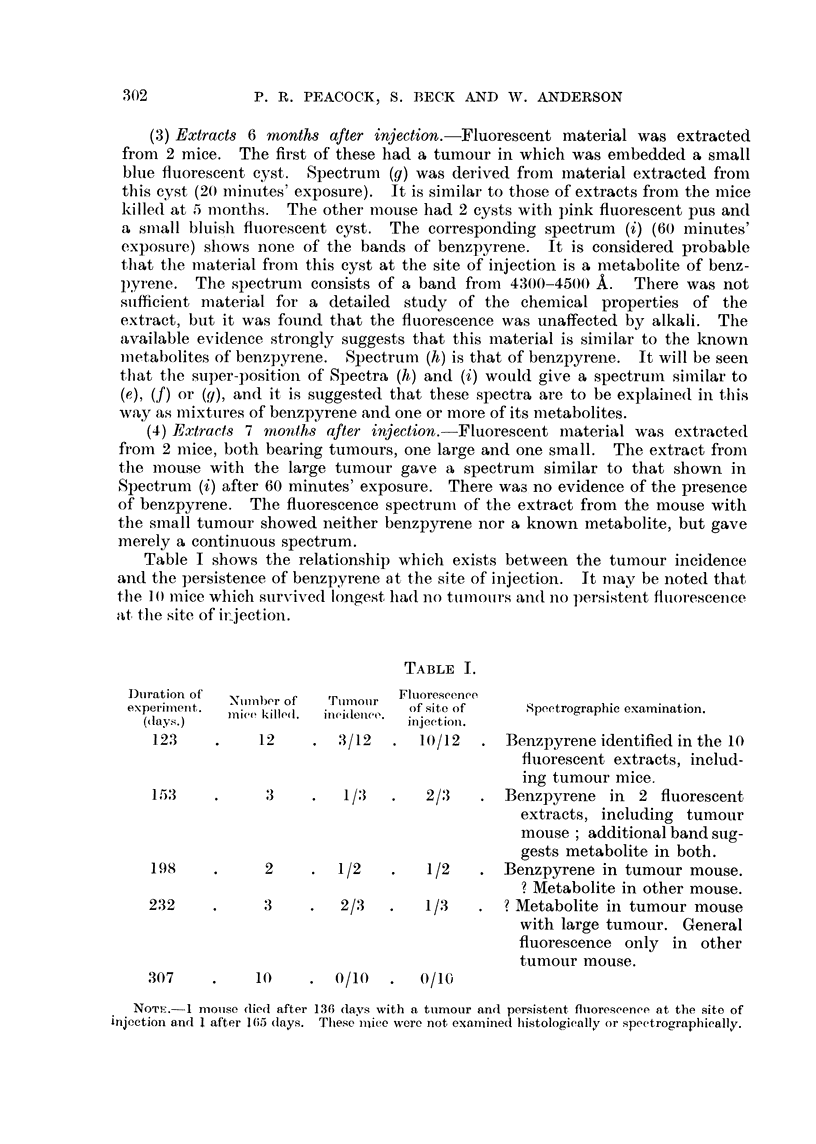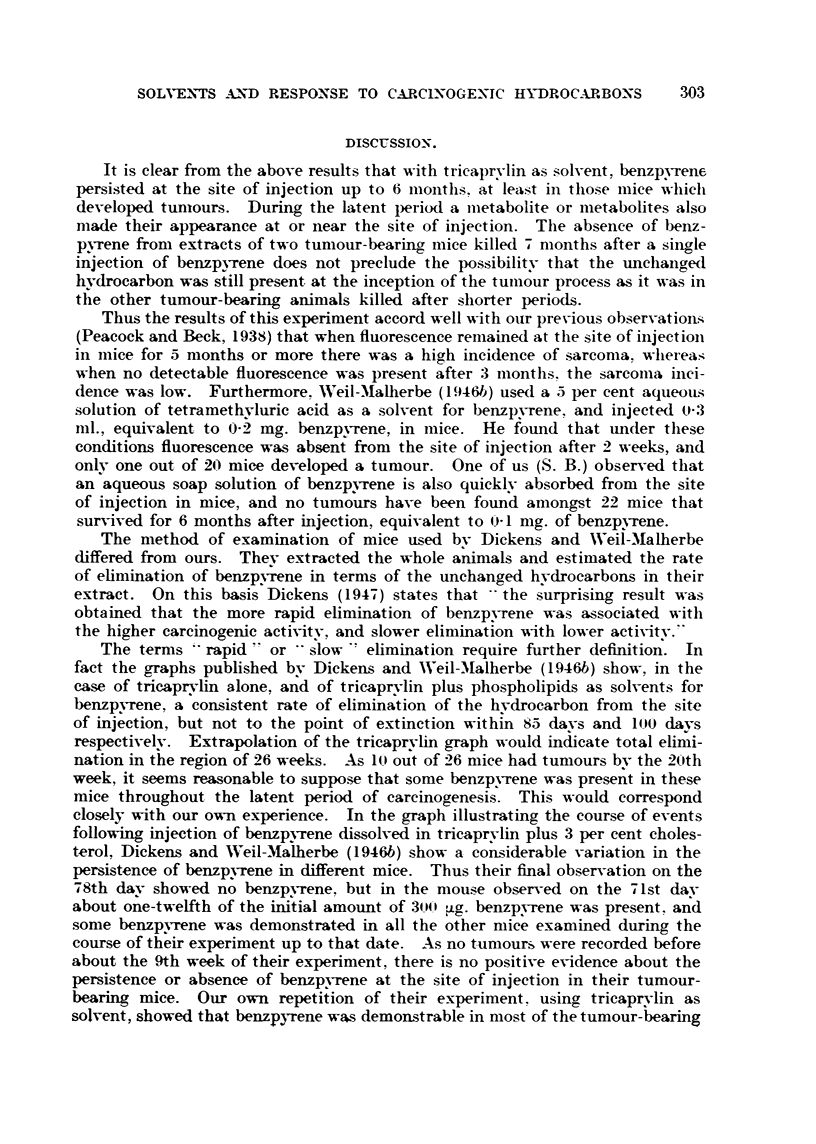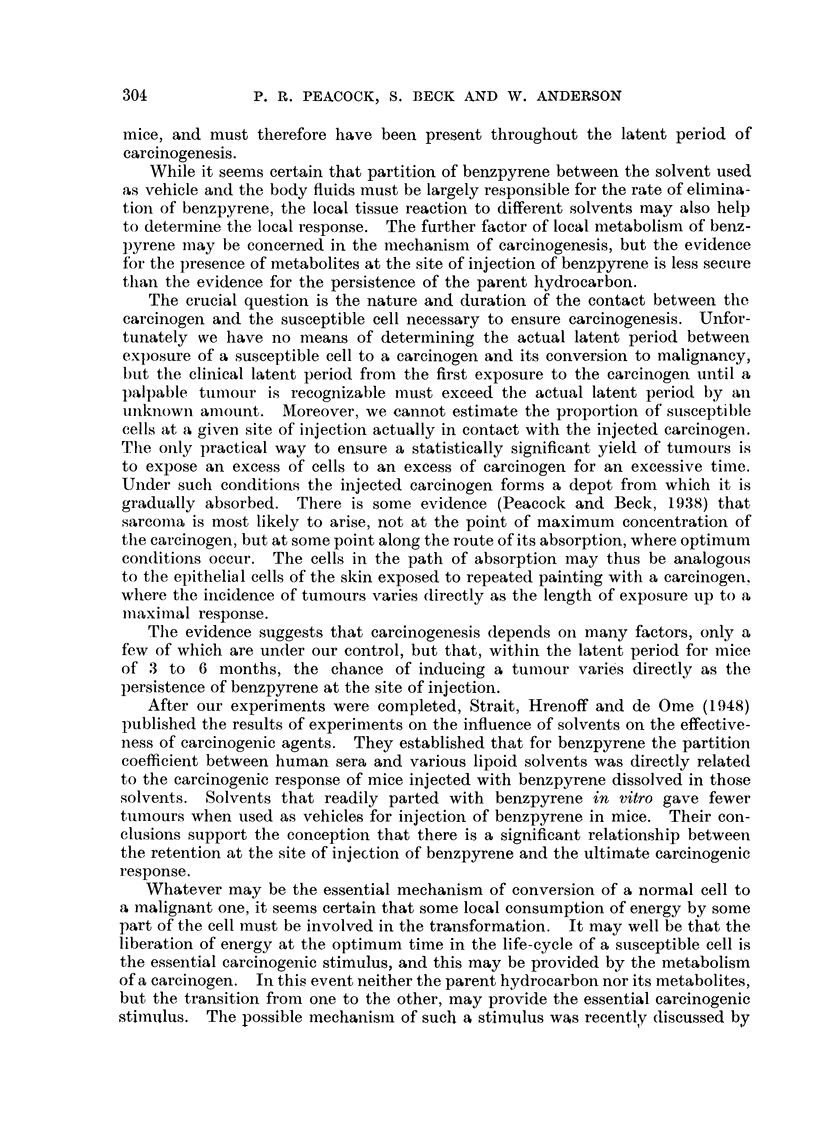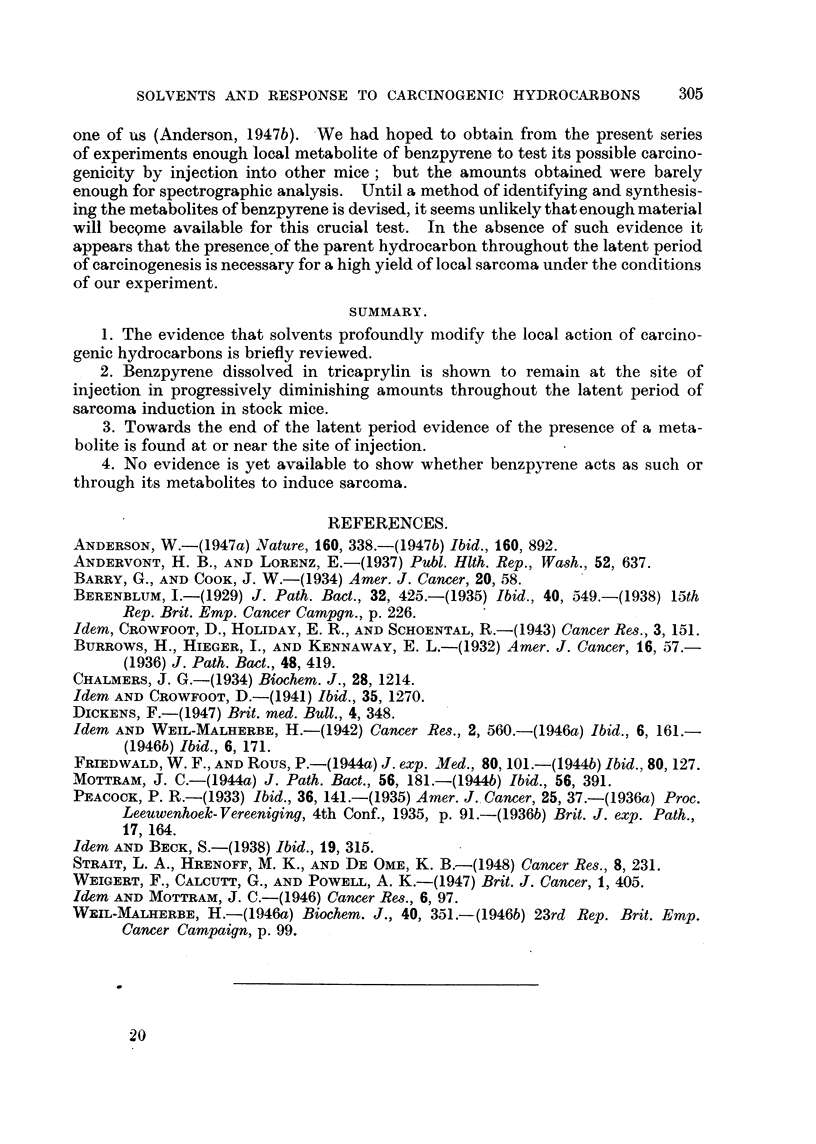# The Influence of Solvents on Tissue Response to Carcinogenic Hydrocarbons

**DOI:** 10.1038/bjc.1949.35

**Published:** 1949-06

**Authors:** P. R. Peacock, S. Beck, W. Anderson

## Abstract

**Images:**


### THE INFLUENCE OF SOLVENTS ON TISSUE RESPONSE TO CARCINOGENIC HYDROCARBONS.

P. R. PEACOCK, S. BECK* AND W. ANDERSON.

*From The Research Department, Glasgow Royal Cancer Hospital.*

Received for publication February 22, 1949.

THE need for controlling the influence of solvents used as vehicles for carcinogenic hydrocarbons was recognized by Burrows, Hieger and Kennaway (1932), who reported experiments designed to test the fat solvents then in use for this purpose, for possible carcinogenic action on the connective tissues of rats and mice. Subsequent work showed that in fact lard, which had been previously heated to 140° C., was a potential carcinogen for the connective tissues of fowls (Peacock, 1933), and that lard alone could give rise to spindle-cell tumours in rats (Barry and Cook, 1934) ; and later that lard, olive oil and other fatty materials could induce connective-tissue tumours, some of which appeared to be malignant in rats, but not in mice, in the experience of Burrows, Hieger and Kennaway (1936).

The first instance of undoubted sarcoma induction at the site of the injection of lard in fowls cited above was complicated by the fact that the same birds had been injected with a solution of 1:2:5:6-dibenzanthracene in lard in the right breast and with lard alone in the left breast. Tumours developed in the right breast only in 7 of these birds, and in both breasts in 3 others. The lard had been heated to 140° C. during the preparation of the material, and cooled to 40° before injection. The following comment is quoted from the above reference : " The possibility that heated lard alone may have determined the occurrence of tumours in a susceptible bird cannot be excluded. . . . Incidentally these experiments suggest that the quantity of dibenzanthracene actually required to initiate sarcomatous growth must be remarkably small, since the bulk of the the injection appears to remain encapsulated throughout the experiment " (Peacock, 1933). The latter assumption subsequently proved to be wrong. To test this point, Chalmers (1934) analysed the contents of encapsulated matter from the site of dibenzanthracene lard injection in one of Peacock's sarcoma-bearing fowls, which died 7 months after the last injection. The method of spectrographic analysis employed was capable of detecting 0·01 mg. of dibenzanthracene in an extract of fowl muscle, but failed to detect dibenzanthracene in the extract of encapsulated debris from this bird.

Further tests on birds in the same experiment confirmed the absence of demonstrable dibenzanthracene 6 months or more after injection. In order to avoid the use of foreign fats, chicken fat and egg yolk fat were used as vehicles for the injection of dibenzanthracene into fowls. It was found that when either

* Working under a full-time grant from the British Empire Cancer Campaign.

of these solvents was used as vehicle dibenzanthracene rapidly disappeared from the site of injection, and was undetectable after 3 weeks. In parallel experiments (Peacock, 1935, 1936*a*) similarly injected birds were kept for longer periods, to test the influence of the solvent on the carcinogenic response. No tumours occurred in 12 birds injected with chicken-fat containing 1–2 mg. dibenzanthracene in 0·4 per cent solution. Moreover, in this group no tissue reaction could be found at the site of injection after a few months, indicating that the homologous fat had caused little disturbance. However, with egg yolk fat as vehicle for dibenzanthracene in 0·4 per cent solution, 2 metastasising sarcomas occurred among 4 birds that survived 9 months or more after injection. Birds injected with yolk fat showed fibrous cysts at the site of injection, similar to those observed in the lard experiment, and in contrast with the absence of such local reaction in birds injected with chicken-fat. There seemed, therefore, to be some aetiological connection between the " foreignness " of the solvent used as vehicle and the local tissue reaction and the ultimate carcinogenic response to dibenzanthracene.

Owing to the costly and time-consuming nature of long-term experiments on fowls work was begun on similar lines on mice. While these experiments were in progress Berenblum (1938) reported the co-carcinogenic action of croton oil. He had previously shown (1929) that mustard gas in dilute solution and (1935) cantharidin had anticarcinogenic properties for the skin of mice, and in the interim had found that no simple relationship between irritant and co- or anti-carcinogenic action was demonstrable. More recently Friedwald and Rous (1944*a*, *b*) reported that chloroform and turpentine both promote the carcinogenic action of methylcholanthrene, and Mottram (1944*a*, *b*) demonstrated that repeated painting with croton oil or resin could elicit a carcinogenic response in mice painted only once with 3:4-benzpyrene.

Thus it is now obvious that solvents may have co- or anti-carcinogenic influence in addition to their capacity as solvents to retain chemical carcinogens at the site of injection for variable periods.

On the basis of the lack of reaction in fowls to injections of chicken fat, we used mouse-fat as a solvent for benzpyrene injected into mice, with the idea of exciting a minimal disturbance due to the solvent. In this species also we observed very little tissue reaction to homologous fat. It seemed likely, therefore, that benzpyrene dissolved in mouse-fat would be readily absorbed along with the fat from the site of injection, and this proved to be the case. In a series of experiments in which various solvents were used as vehicles for benzpyrene it was found that those solvents which remained encapsulated at the site of injection, e.g. olive oil, tended to retain the dissolved benzpyrene as judged by the persistence of violet fluorescence, whereas in the case of those that were rapidly absorbed, e.g. mouse fat or ether, fluorescence could not be detected after a few weeks (Peacock and Beck, 1938). Moreover, in the former group the incidence of sarcomas was high, and in the latter group it was low. This suggested that there may be an optimal period for the retention of a carcinogen at the site of injection for the induction of a tumour, and that in mice this period was about 5 months.

Dickens and Weil-Malherbe (1942) at first confirmed our observation with mouse fat, but later (1946*a*), as a result of intensive experiments, found that the effect was not constant and was related to the phospho-lipid content of the solvent. Fats with a high phospho-lipid content seemed to inhibit carcinogenic action, while those with a high cholesterol content seemed to enhance it. To obviate variations between samples of fat of animal or vegetable origin, Dickens and Weil-Malherbe adopted tricaprylin as a solvent for benzpyrene in further experiments. They also reported the interesting fact that certain purine compounds are good solvents for hydrocarbons giving aqueous solutions (Weil-Malherbe, 1946*a*).

It had already been shown by Andervont and Lorenz (1937) that serum was a solvent for benzpyrene and other hydrocarbons.

The solvent capacity of the circulating blood must thus be a factor in determining the localization in, or elimination of hydrocarbons from, the site of their injection; but there is, as Dickens pointed out (1947), a distinction to be made between elimination of unchanged hydrocarbon and local metabolism, processes which might elicit very different local tissue reactions, though either would reduce the amount of detectable hydrocarbon at the site of injection.

It still remained to be shown whether or not the fluorescence at the site of injection of benzpyrene (Peacock and Beck, 1938) throughout the latent period of carcinogenesis was due to the unchanged hydrocarbon. In the experiment now reported tricaprylin was used as vehicle for the injection of benzpyrene, as advocated by Dickens and Weil-Malherbe.

### EXPERIMENTAL.

Thirty-six mice of mixed stock were each injected subcutaneously in the right flank with 0·3 ml. of a 0·1 per cent solution of benzpyrene in tricaprylin ; 3:4-benzpyrene was supplied by Ward, Blenkinsop & Co., Ltd., London, and tricaprylin by British Drug Houses, Ltd. The mice were killed after different periods (Table I), the subcutaneous tissues exposed and examined for ultra-violet fluorescence. Sixteen of 20 mice killed at various times over a period of 7 months had fluorescent material located at the original site of injection ; ulceration and sepsis at the site of injection killed 4 mice early in the experiment. In 3 of these mice no sign of the injected material was found, probably due to sloughing and leakage from the site of injection. The fourth had a small spot of fluorescence. These mice were not examined further ; in the remainder the fluorescent material was extracted with benzene, with the exception of 2 mice, which died after 4½ and 5½ months, both showing persistent fluorescence and tumours at the site of injection.

After the third month of experiment tumours began to be clinically recognized at the sites of injection. As most of these tumours were similar to many such induced sarcomas in mice, not all were histologically examined. One tumour in the abdominal cavity associated with intraperitoneal deposits of fluorescent material was probably induced by benzpyrene injected accidentally through the abdominal wall. This tumour was a pleomorphic sarcoma. Another tumour occurred in an ulcerated area at the site of injection. This proved to be a squamous carcinoma. Of the remaining tumours the only one examined histologically was the familiar type of spindle-cell sarcoma.

As far as the injected benzpyrene was concerned the gross appearances in normal and ultraviolet light varied considerably in different mice, and the preliminary method of extraction was modified to suit the local condition. In most cases one or more small fluorescent cysts were present, and these were opened and their contents expressed into 2 ml. benzene, yielding a fluorescent solution. In other mice numerous minute spots of brightly fluorescent material pervading a tumour or merely a general fluorescence localized in the neighbourhood of the site of injection were found. The region of tissue bearing the fluorescent material was dissected out in order to minimize the extraction of normal or adventitious fluorescent material which might mask the fluorescence of small traces of the carcinogen. In these cases the fluorescent tissues were triturated in benzene with a glass rod, or if this was unsuccessful the tissue was hydrolysed with 10 per cent aqueous alcoholic potash (50 ml. EtOH to 50 ml. $H_2O$), the alcohol removed, the unsaponifiable fraction extracted with 5 ml. benzene, and the benzene extract dried over anhydrous sodium sulphate.

Since the main object of these experiments was to demonstrate the presence or absence of benzpyrene after various periods, the experiments were not conducted quantitatively. However, the limit of sensitivity of the method of detection provides some indication of the quantities involved (*vide infra*). To eliminate the possibility of a false positive result due to traces of benzpyrene adhering to the apparatus used in the experiments, benzene washings of each piece of chemical apparatus or other instrument used, were examined under the ultraviolet lamp immediately before its use. Attention was drawn to the necessity for such precautions recently (Anderson, 1947*a*). Owing to the difficulty of eliminating minute traces of material from apparatus, such traces may be recorded by the fluorescence method of detection, and lead to a false positive result. The benzene (British Drug Houses, Ltd., AR.) used was rectified before use and was free from visible fluorescence. It is considered that the precautions employed exclude the possibility of any false positive result.

The fluorescence spectra were recorded with a Hilger Medium Quartz Spectrograph, using Ilford Iso-Zenith Plates. A small glass cell of 0·5 ml. capacity held the solution for test close to the slit of the spectrograph, and the fluorescence was excited by radiation from a G.E.C. Osira lamp focused on to the cell by a condensing lens. The slit width used throughout was 0·1 mm. and the exposures varied from 1–60 minutes, depending on the fluorescence intensity of the solution.

### RESULTS.

*The nature of the fluorescent material at the site of injection.*

The relative intensities of the characteristic bands of the fluorescence spectrum of benzpyrene in pure benzene (Fig. 1, Spectrum (*a*)) are dependent on the sensitivity of the photographic emulsion to the different wavelengths. With the apparatus used in these experiments it is possible to obtain a good spectrum of benzpyrene at a concentration of 0·001 per cent with an exposure of 5 minutes, while if a 60 minutes' exposure is used it is just possible to detect the two strong groups of bands at 4030–4120Å and 4260–4400Å at a concentration of 1 $\mu$g./100 ml. This represents the practical limit of the technique, for it is just possible to detect fluorescence in a solution of this concentration by visual inspection. The volume of solution used in the test is 0·5 ml., representing 0·005 $\mu$g. benzpyrene at the limiting concentration.

Detection of benzpyrene in tissues examined by the technique described above implies that at least 0·02 $\mu$g. benzpyrene was present. In fact, however, the quantities of benzpyrene present at the sites of injection must have greatly exceeded this value for several reasons : (1) No attempt was made to extract all the fluorescent material present at the site of injection. (2) The exposures required to demonstrate benzpyrene were under 60 minutes in every case. (3)

FIG. 1.—Fluorescence spectra of 3:4-benzpyrene and metabolites.

In all these cases the benzpyrene bands were very much stronger than those obtained with a solution of concentration 1 μg./100 ml. benzpyrene (limit of sensitivity of the method).

In long exposures reflection of mercury lines is recorded along with the fluorescence spectrum of the extract under examination. This is not a disadvantage, as it is possible to distinguish between the sharp lines of the mercury spectrum and the characteristic groups of bands due to the fluorescence of benzpyrene. The mercury lines accurately check the wave-length scale.

Spectrum (*b*) shows the weak mercury line at 4040 Å and 4070 Å in approximately the same region as the strong fluorescent bands of benzpyrene at 4030–4120 Å. The strong mercury line 3650 Å stands out clearly in the near-ultraviolet region ; the latter line can be seen in all fluorescent spectra taken with this equipment, but the weaker mercury lines in the visible spectrum are only obvious after long exposures.

Spectrum (*c*) exposure 20 minutes shows the fluorescence of a metabolite of benzpyrene extracted from the faeces of rats fed with benzpyrene dissolved in milk, and corresponds to the fluorescent derivative of benzpyrene detected by Peacock (1936*b*) in the bile of rabbits, guinea-pigs and fowls after intravenous injection of benzpyrene colloid. It was later deduced that this derivative was a monohydroxy benzpyrene (Chalmers and Crowfoot, 1941), and further work indicated that it was 8-hydroxy-benzpyrene (Berenblum, Crowfoot, Holiday and Schoental, 1943). Weigert and Mottram (1946) have pointed out that the formation of a monohydroxy benzpyrene may be preceded by the production of a diol, and have published fluorescence spectrograms of fractions extracted from tissues exposed to benzpyrene in which the main fluorescence is in the region 4200–4600 Å. Weigert, Calcutt and Powell (1947) showed that in mouse skin painted with benzpyrene the only detectable metabolite over periods up to 24 hours is that corresponding to Weigert and Mottram's $BPX_2$ represented by them by the following formula :

where $R_1$ and $R_2$ are unidentified cell constituents.

The different fluorescence spectra encountered in the present investigation are now considered in relation to the length of time after injection.

(1) *Extracts made 4 months after injection.*—Fluorescent material was extracted from 10 mice. A suitable spectrum was obtained from all of these, with exposures of 5–10 minutes—a representative spectrum is reproduced as Spectrum (*d*). All the spectra were the same, and are identical with that of benzpyrene (Spectrum (*a*)).

(2) *Extracts 5 months after injection.*—Fluorescent material was extracted from 2 mice. Spectra (*e*) and (*f*) correspond to these extracts. Both spectra have the benzpyrene bands at 4030–4120 Å, but the fluorescence also extends over the region 4260–4600 Å, which coincides with the longer wave-length region of the benzpyrene spectrum and also with the range of the benzpyrene metabolites. The interesting feature of these spectra is that they show an increase in the intensity of the fluorescence in the latter region, compared with the spectrum of unchanged benzpyrene. These spectra are interpreted as a mixture of the spectra of benzpyrene and another material with a spectrum similar to that of the fluorescent metabolites of benzpyrene.

(3) *Extracts 6 months after injection.*—Fluorescent material was extracted from 2 mice. The first of these had a tumour in which was embedded a small blue fluorescent cyst. Spectrum (*g*) was derived from material extracted from this cyst (20 minutes' exposure). It is similar to those of extracts from the mice killed at 5 months. The other mouse had 2 cysts with pink fluorescent pus and a small bluish fluorescent cyst. The corresponding spectrum (*i*) (60 minutes' exposure) shows none of the bands of benzpyrene. It is considered probable that the material from this cyst at the site of injection is a metabolite of benzpyrene. The spectrum consists of a band from 4300–4500 Å. There was not sufficient material for a detailed study of the chemical properties of the extract, but it was found that the fluorescence was unaffected by alkali. The available evidence strongly suggests that this material is similar to the known metabolites of benzpyrene. Spectrum (*h*) is that of benzpyrene. It will be seen that the super-position of Spectra (*h*) and (*i*) would give a spectrum similar to (*e*), (*f*) or (*g*), and it is suggested that these spectra are to be explained in this way as mixtures of benzpyrene and one or more of its metabolites.

(4) *Extracts 7 months after injection.*—Fluorescent material was extracted from 2 mice, both bearing tumours, one large and one small. The extract from the mouse with the large tumour gave a spectrum similar to that shown in Spectrum (*i*) after 60 minutes' exposure. There was no evidence of the presence of benzpyrene. The fluorescence spectrum of the extract from the mouse with the small tumour showed neither benzpyrene nor a known metabolite, but gave merely a continuous spectrum.

Table I shows the relationship which exists between the tumour incidence and the persistence of benzpyrene at the site of injection. It may be noted that the 10 mice which survived longest had no tumours and no persistent fluorescence at the site of injection.

TABLE I.

| Duration of experiment. (days.) | Number of mice killed. | Tumour incidence. | Fluorescence of site of injection. | Spectrographic examination. |
|---|---|---|---|---|
| 123 | 12 | 3/12 | 10/12 | Benzpyrene identified in the 10 fluorescent extracts, including tumour mice. |
| 153 | 3 | 1/3 | 2/3 | Benzpyrene in 2 fluorescent extracts, including tumour mouse ; additional band suggests metabolite in both. |
| 198 | 2 | 1/2 | 1/2 | Benzpyrene in tumour mouse. ? Metabolite in other mouse. |
| 232 | 3 | 2/3 | 1/3 | ? Metabolite in tumour mouse with large tumour. General fluorescence only in other tumour mouse. |
| 307 | 10 | 0/10 | 0/10 | |

NOTE.—1 mouse died after 136 days with a tumour and persistent fluorescence at the site of injection and 1 after 165 days. These mice were not examined histologically or spectrographically.

### DISCUSSION.

It is clear from the above results that with tricaprylin as solvent, benzpyrene persisted at the site of injection up to 6 months, at least in those mice which developed tumours. During the latent period a metabolite or metabolites also made their appearance at or near the site of injection. The absence of benzpyrene from extracts of two tumour-bearing mice killed 7 months after a single injection of benzpyrene does not preclude the possibility that the unchanged hydrocarbon was still present at the inception of the tumour process as it was in the other tumour-bearing animals killed after shorter periods.

Thus the results of this experiment accord well with our previous observations (Peacock and Beck, 1938) that when fluorescence remained at the site of injection in mice for 5 months or more there was a high incidence of sarcoma, whereas when no detectable fluorescence was present after 3 months, the sarcoma incidence was low. Furthermore, Weil-Malherbe (1946*b*) used a 5 per cent aqueous solution of tetramethyluric acid as a solvent for benzpyrene, and injected 0·3 ml., equivalent to 0·2 mg. benzpyrene, in mice. He found that under these conditions fluorescence was absent from the site of injection after 2 weeks, and only one out of 20 mice developed a tumour. One of us (S. B.) observed that an aqueous soap solution of benzpyrene is also quickly absorbed from the site of injection in mice, and no tumours have been found amongst 22 mice that survived for 6 months after injection, equivalent to 0·1 mg. of benzpyrene.

The method of examination of mice used by Dickens and Weil-Malherbe differed from ours. They extracted the whole animals and estimated the rate of elimination of benzpyrene in terms of the unchanged hydrocarbons in their extract. On this basis Dickens (1947) states that "the surprising result was obtained that the more rapid elimination of benzpyrene was associated with the higher carcinogenic activity, and slower elimination with lower activity."

The terms "rapid" or "slow" elimination require further definition. In fact the graphs published by Dickens and Weil-Malherbe (1946*b*) show, in the case of tricaprylin alone, and of tricaprylin plus phospholipids as solvents for benzpyrene, a consistent rate of elimination of the hydrocarbon from the site of injection, but not to the point of extinction within 85 days and 100 days respectively. Extrapolation of the tricaprylin graph would indicate total elimination in the region of 26 weeks. As 10 out of 26 mice had tumours by the 20th week, it seems reasonable to suppose that some benzpyrene was present in these mice throughout the latent period of carcinogenesis. This would correspond closely with our own experience. In the graph illustrating the course of events following injection of benzpyrene dissolved in tricaprylin plus 3 per cent cholesterol, Dickens and Weil-Malherbe (1946*b*) show a considerable variation in the persistence of benzpyrene in different mice. Thus their final observation on the 78th day showed no benzpyrene, but in the mouse observed on the 71st day about one-twelfth of the initial amount of 300 $\mu$g. benzpyrene was present, and some benzpyrene was demonstrated in all the other mice examined during the course of their experiment up to that date. As no tumours were recorded before about the 9th week of their experiment, there is no positive evidence about the persistence or absence of benzpyrene at the site of injection in their tumour-bearing mice. Our own repetition of their experiment, using tricaprylin as solvent, showed that benzpyrene was demonstrable in most of the tumour-bearing mice, and must therefore have been present throughout the latent period of carcinogenesis.

While it seems certain that partition of benzpyrene between the solvent used as vehicle and the body fluids must be largely responsible for the rate of elimination of benzpyrene, the local tissue reaction to different solvents may also help to determine the local response. The further factor of local metabolism of benzpyrene may be concerned in the mechanism of carcinogenesis, but the evidence for the presence of metabolites at the site of injection of benzpyrene is less secure than the evidence for the persistence of the parent hydrocarbon.

The crucial question is the nature and duration of the contact between the carcinogen and the susceptible cell necessary to ensure carcinogenesis. Unfortunately we have no means of determining the actual latent period between exposure of a susceptible cell to a carcinogen and its conversion to malignancy, but the clinical latent period from the first exposure to the carcinogen until a palpable tumour is recognizable must exceed the actual latent period by an unknown amount. Moreover, we cannot estimate the proportion of susceptible cells at a given site of injection actually in contact with the injected carcinogen. The only practical way to ensure a statistically significant yield of tumours is to expose an excess of cells to an excess of carcinogen for an excessive time. Under such conditions the injected carcinogen forms a depot from which it is gradually absorbed. There is some evidence (Peacock and Beck, 1938) that sarcoma is most likely to arise, not at the point of maximum concentration of the carcinogen, but at some point along the route of its absorption, where optimum conditions occur. The cells in the path of absorption may thus be analogous to the epithelial cells of the skin exposed to repeated painting with a carcinogen, where the incidence of tumours varies directly as the length of exposure up to a maximal response.

The evidence suggests that carcinogenesis depends on many factors, only a few of which are under our control, but that, within the latent period for mice of 3 to 6 months, the chance of inducing a tumour varies directly as the persistence of benzpyrene at the site of injection.

After our experiments were completed, Strait, Hrenoff and de Ome (1948) published the results of experiments on the influence of solvents on the effectiveness of carcinogenic agents. They established that for benzpyrene the partition coefficient between human sera and various lipoid solvents was directly related to the carcinogenic response of mice injected with benzpyrene dissolved in those solvents. Solvents that readily parted with benzpyrene *in vitro* gave fewer tumours when used as vehicles for injection of benzpyrene in mice. Their conclusions support the conception that there is a significant relationship between the retention at the site of injection of benzpyrene and the ultimate carcinogenic response.

Whatever may be the essential mechanism of conversion of a normal cell to a malignant one, it seems certain that some local consumption of energy by some part of the cell must be involved in the transformation. It may well be that the liberation of energy at the optimum time in the life-cycle of a susceptible cell is the essential carcinogenic stimulus, and this may be provided by the metabolism of a carcinogen. In this event neither the parent hydrocarbon nor its metabolites, but the transition from one to the other, may provide the essential carcinogenic stimulus. The possible mechanism of such a stimulus was recently discussed by one of us (Anderson, 1947*b*). We had hoped to obtain from the present series of experiments enough local metabolite of benzpyrene to test its possible carcinogenicity by injection into other mice ; but the amounts obtained were barely enough for spectrographic analysis. Until a method of identifying and synthesising the metabolites of benzpyrene is devised, it seems unlikely that enough material will become available for this crucial test. In the absence of such evidence it appears that the presence of the parent hydrocarbon throughout the latent period of carcinogenesis is necessary for a high yield of local sarcoma under the conditions of our experiment.

SUMMARY.

1. The evidence that solvents profoundly modify the local action of carcinogenic hydrocarbons is briefly reviewed.
2. Benzpyrene dissolved in tricaprylin is shown to remain at the site of injection in progressively diminishing amounts throughout the latent period of sarcoma induction in stock mice.
3. Towards the end of the latent period evidence of the presence of a metabolite is found at or near the site of injection.
4. No evidence is yet available to show whether benzpyrene acts as such or through its metabolites to induce sarcoma.

### REFERENCES.

ANDERSON, W.—(1947*a*) *Nature*, **160**, 338.—(1947*b*) *Ibid.*, **160**, 892.
ANDERVONT, H. B., AND LORENZ, E.—(1937) *Publ. Hlth. Rep., Wash.*, **52**, 637.
BARRY, G., AND COOK, J. W.—(1934) *Amer. J. Cancer*, **20**, 58.
BERENBLUM, I.—(1929) *J. Path. Bact.*, **32**, 425.—(1935) *Ibid.*, **40**, 549.—(1938) *15th Rep. Brit. Emp. Cancer Campgn.*, p. 226.
*Idem*, CROWFOOT, D., HOLIDAY, E. R., AND SCHOENTAL, R.—(1943) *Cancer Res.*, **3**, 151.
BURROWS, H., HIEGER, I., AND KENNAWAY, E. L.—(1932) *Amer. J. Cancer*, **16**, 57.—(1936) *J. Path. Bact.*, **48**, 419.
CHALMERS, J. G.—(1934) *Biochem. J.*, **28**, 1214.
*Idem* AND CROWFOOT, D.—(1941) *Ibid.*, **35**, 1270.
DICKENS, F.—(1947) *Brit. med. Bull.*, **4**, 348.
*Idem* AND WEIL-MALHERBE, H.—(1942) *Cancer Res.*, **2**, 560.—(1946*a*) *Ibid.*, **6**, 161.—(1946*b*) *Ibid.*, **6**, 171.
FRIEDWALD, W. F., AND ROUS, P.—(1944*a*) *J. exp. Med.*, **80**, 101.—(1944*b*) *Ibid.*, **80**, 127.
MOTTRAM, J. C.—(1944*a*) *J. Path. Bact.*, **56**, 181.—(1944*b*) *Ibid.*, **56**, 391.
PEACOCK, P. R.—(1933) *Ibid.*, **36**, 141.—(1935) *Amer. J. Cancer*, **25**, 37.—(1936*a*) *Proc. Leeuwenhoek-Vereeniging*, 4th Conf., 1935, p. 91.—(1936*b*) *Brit. J. exp. Path.*, **17**, 164.
*Idem* AND BECK, S.—(1938) *Ibid.*, **19**, 315.
STRAIT, L. A., HRENOFF, M. K., AND DE OME, K. B.—(1948) *Cancer Res.*, **8**, 231.
WEIGERT, F., CALCUTT, G., AND POWELL, A. K.—(1947) *Brit. J. Cancer*, **1**, 405.
*Idem* AND MOTTRAM, J. C.—(1946) *Cancer Res.*, **6**, 97.
WEIL-MALHERBE, H.—(1946*a*) *Biochem. J.*, **40**, 351.—(1946*b*) *23rd Rep. Brit. Emp. Cancer Campaign*, p. 99.